# Epidemiological and Virological Characteristics of Pandemic Influenza A (H1N1) School Outbreaks in China in 2009

**DOI:** 10.1371/journal.pone.0045898

**Published:** 2012-09-27

**Authors:** Lei Yan, Yan Gao, Yong Zhang, Michael Tildesley, Liqi Liu, Ye Zhang, Leying Wen, Wei Wang, Xiaodan Li, Ying Hu, Tian Bai, Min Wang, Yuhong Zeng, Dingming Wang, Xianjun Wang, Yu Lan, Shiwen Wang, Yuelong Shu

**Affiliations:** 1 State Key Laboratory for Molecular Virology and Genetic Engineering, National Institute for Viral Disease Control and Prevention, Chinese Center for Disease Control and Prevention, Beijing, China; 2 School of Mathematical Sciences, Beijing Normal University, Beijing, China; 3 Centre for Complexity Science, Zeeman Building, University of Warwick, Coventry, United Kingdom; 4 US National Institutes of Health, Fogarty International Center, Bethesda, Maryland, United States of America; 5 Center for Disease Control and Prevention of Guizhou Province, GuiYang, China; 6 Center for Disease Control and Prevention of Shandong Province, Jinan, China; Institut Pasteur of Shanghai, Chinese Academy of Sciences, China

## Abstract

**Background:**

During the 2009 pandemic influenza H1N1 (2009) virus (pH1N1) outbreak, school students were at an increased risk of infection by the pH1N1 virus. However, the estimation of the attack rate showed significant variability.

**Methods:**

Two school outbreaks were investigated in this study. A questionnaire was designed to collect information by interview. Throat samples were collected from all the subjects in this study 6 times and sero samples 3 times to confirm the infection and to determine viral shedding. Data analysis was performed using the software STATA 9.0.

**Findings:**

The attack rate of the pH1N1 outbreak was 58.3% for the primary school, and 52.9% for the middle school. The asymptomatic infection rates of the two schools were 35.8% and 37.6% respectively. Peak virus shedding occurred on the day of ARI symptoms onset, followed by a steady decrease over subsequent days (p = 0.026). No difference was found either in viral shedding or HI titer between the symptomatic and the asymptomatic infectious groups.

**Conclusions:**

School children were found to be at a high risk of infection by the novel virus. This may be because of a heightened risk of transmission owing to increased mixing at boarding school, or a lack of immunity owing to socio-economic status. We conclude that asymptomatically infectious cases may play an important role in transmission of the pH1N1 virus.

## Introduction

During April–May 2009, after early outbreaks in North America in April 2009, the pandemic influenza A/H1N1 virus spread rapidly around the world [1], [2]. The World Health Organization (WHO) declared a pandemic in June 2009 [3], which lasted until August 10, 2009 [4]. During the post-pandemic period [5], the H1N1 virus continued to circulate, mirroring the behavior of the seasonal influenza virus.

During 2009, outbreaks of H1N1 were reported in many countries around the world [6], [7], [8]. Early data suggested that most of the deaths caused by pandemic influenza occurred in younger people, including those who were otherwise healthy [9]. Pregnant women, younger children and people of any age with certain chronic lung or other medical conditions appeared to be at higher risk of more complicated or severe illness [10], [11]. However, some reports showed that the majority of people with pandemic influenza experienced mild illness and the overall risks of dying from this infection were low. After the peak of a second wave of infection in Pittsburgh, the seroprevalence of hemagglutination-inhibition (HI) antibody suggested that about 21% of all individuals and 45% of those between the ages of 10 and 19 years had become infected [12].

Subsequent reports suggested that the actual prevalence had been largely underestimated, whilst the symptomatic severity had been overestimated, and that serological investigation may have been helpful in establishing a more accurate estimate of the infection rate [5], [13].

Two influenza outbreaks attributed to the pH1N1 virus were identified in a middle school (age range of 11–15 years old) in Shandong province in northern China and a primary school (age range of 6–15 years old) in Guizhou province in southern China respectively in late November 2009. The schools remained open and no drug intervention was used in an attempt to control the spread of the disease. No other non-medical interventions were used – there was no school doctor on site at either school and any student that had fever(>37°C) was isolated at home. Any further information on medical interventions for any student sent home was not collected. As a part of this study, we monitored both schools for one month in order to ascertain the epidemiological and virological characteristics of both outbreaks. A questionnaire was carried out to collect demographic and medical information to describe the symptoms of all infected cases. Infection was confirmed by real-time RT-PCR(rRT-PCR) and/or HI. Viral shedding was tested with the serial swabs by quantitative real-time PCR.

## Results

### Attack Rate

**Figure 1:**
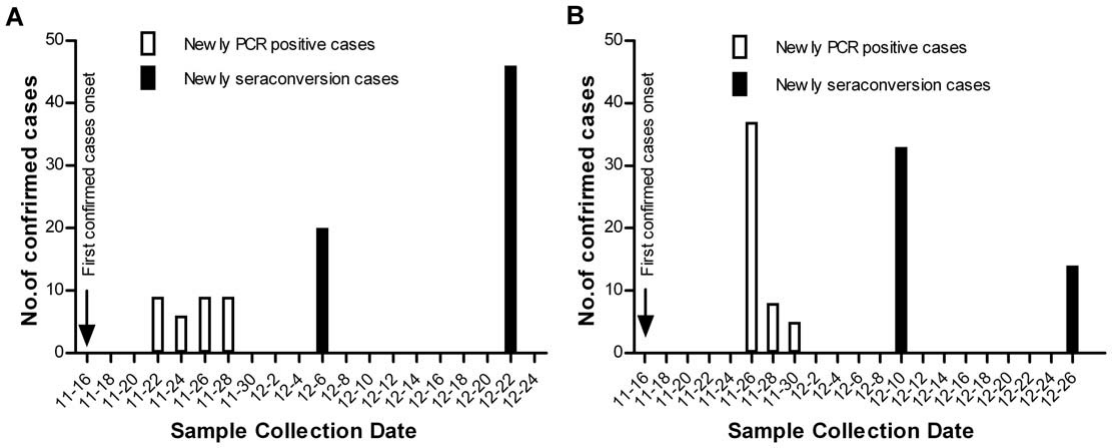
Number of newly confirmed cases by rRT-PCR or HI in the middle school and the primary school. (A) The arrow indicates the date of onset of the first confirmed case in the middle school. The solid white bars represent the number of newly confirmed cases by rRT-PCR. The solid black bars represent the number of newly confirmed cases by HI. (B) The arrow indicates the date of onset of the first confirmed case in the primary school. The solid white bars represent the number of newly confirmed cases by rRT-PCR. The solid black bars represent the number of newly confirmed cases by HI.

In the middle school, amongst the 214 students who answered the questionnaires, one hundred students were confirmed to have the pH1N1 infection by HI and/or rRT-PCR, and the attack rate was 52.9% (95%CI, 45.8% to 60.0%). Samples from 25 students were incomplete and therefore excluded from this analysis. At the beginning of the study (Nov. 24), one sample was found to have HI titer≥40 (the baseline HI titer was set as 5). Sero samples of 66 students subsequently converted positive. In those cases, the first sero sample was negative, whilst the second or the third sample converted to positive with a titer four-fold higher than the initial sample. The seroconversion rate was 39.8% (95%CI, 31.0% to 48.7%). 56 students’ throat swab samples were detected positive at least once by rRT-PCR (26.2%, 95%CI, 23.2% to 32.1%) (Figure 1).

In the primary school, at the beginning of the study (on Nov. 26) 17 samples were found to be positive by HI, and sero samples of 47 students subsequently converted positive. The seroconversion rate was 35.3% (95%CI, 26.7% to 43.0%). 51 students’ swab samples (32.7%, 95%CI, 25.3% to 40.1%) were detected positive at least once by rRT-PCR (Figure 1). Amongst the 156 subjects, a total of 70 students were confirmed to be infected with the H1N1 virus, and the attack rate was 58.3% (95%CI, 49.5% to 67.2%). Samples from 36 students were incomplete and therefore excluded from this analysis.

### Asymptomatic Infection Rate

In this study, only some confirmed cases showed significant symptoms (Table S1). Amongst the 189 students in the middle school from whom sero and throat swabs samples were collected, 100 cases were confirmed by HI and/or rRT-PCR test, whilst 71 cases were reported without typical ARI symptoms. Here we defined symptomatic infection as a confirmed infection with ARI symptoms, and an asymptomatic infection as confirmed infection without ARI symptoms. The asymptomatic infection rate was 37.6% (71/189, 95%CI:30.7% to 44.5%). Amongst the 120 students in the primary school, 70 cases were confirmed by HI and/or rRT-PCR test, whilst 43 cases were reported without typical ARI symptoms. The asymptomatic infection rate was 35.8% (43/120, 95%CI, 27.3% to 44.4%). The asymptomatic infection rate of the primary school was found to be not significantly different from that of the middle school (p = 0.758).

### Viral Shedding

We detected viral shedding with Acute Respiratory Illness (ARI) symptoms in 45 specimens (see Materials and Methods). The median ratio of pandemic H1N1 copy to β-actin was 1.6 (ranging from 0.0 to 230.7). Initial viral shedding was detected 2 days before the onset of ARI symptoms and the majority of shedding occurred 0–5 days after the onset of ARI symptoms. Peak virus shedding for pH1N1 infection occurred on the day of the onset of ARI symptoms (max ratio = 230.7), followed by a steady decrease over the subsequent 8 days (p = 0.026, R^2^ = 0.18).

### Comparison between Symptomatic and Asymptomatic Infection Groups on rRT-PCR Test, Viral Loads, and HI Test

In the middle school, the percentage of rRT-PCR positive confirmed cases amongst the symptomatic infection group was 58.6%(17/29), whilst that of the asymptomatic infection group was 54.9% (39/71). In the primary school, 77.8% (21/27) of symptomatic infections were rRT-PCR positive, whilst 69.8% (30/43) of asymptomatic infections were rRT-PCR positive. The rRT-PCR positive percentage in the symptomatic infection group was not significantly different from that of the asymptomatic infection group in both the middle school and the primary school (Table 1).

**Table 1:** Table showing the number of rRT-PCR confirmed cases for symptomatic and asymptomatic infection groups for the middle school in Shandong and the primary school in Guizhou.

Location	Result	Symptomatic infection	Asymptomatic infection	P value
Shandong	Positive	17 (30.36%)	39 (34.21%)	0.736
	Negative	12 (21.43%)	32 (28.07%)	
Guizhou	Positive	21 (37.50%)	30 (26.32%)	0.463
	Negative	6 (10.71%)	13 (11.40%)	
	Total	56	114	

Viral loads of the pandemic H1N1 virus were not found to differ significantly between the symptomatic and asymptomatic infection groups in both the middle school and the primary school. In the middle school, the mean viral load (ratio of the copy of M gene of influenza A virus to the copy of β-actin gene of the host cell) was found to be 38.9 in the symptomatic infection group and 66.5 in the asymptomatic infection group. In the primary school in Guizhou, the mean viral load was found to be 7.9 and 5.6 in the symptomatic and asymptomatic infection groups respectively. The viral loads in the symptomatic infection group were not significantly different from that of the asymptomatic infection group in both the middle school and the primary school (Table 2).

**Table 2:** Table showing mean viral loads for rRT-PCR confirmed symptomatic and asymptomatic cases in the middle school in Shandong and the primary school in Guizhou.

Location	Case number	Mean viral load amongstSymptomatic cases*	Mean viral load amongst Asymptomatic cases*	P value
Shandong	38 (33.04%)	38.9	66.5	0.594
Guizhou	77 (66.96%)	7.9	5.6	0.422
Total	115	17.8	26.2	0.63

* pH1N1 copies per β-actin.

In the middle school, the HI conversion rate was 74.1%(20/27) in the symptomatic infection group and 70.8% (46/65) in the asymptomatic infection group. In the primary school, the HI conversion rate was 69.2% (18/26) and 69.0% (29/42) in the symptomatic and asymptomatic infection groups respectively. HI conversion rate in both schools was found to not differ significantly between the symptomatic and asymptomatic infection groups (Table 3).

**Table 3:** HI confirmed cases for symptomatic and asymptomatic infection groups for the middle school in Shandong and the primary school in Guizhou.

Location	Results	Symptomatic (%)	Asymptomatic (%)	P value
Shandong	Positive	20 (37.74%)	46 (42.99%)	0.749
	Negative	7 (13.21%)	19 (17.76%)	
Guizhou	Positive	18 (33.96%)	29 (27.10%)	0.987
	Negative	8 (15.09%)	13 (12.15%)	
	Total	53	107	

### Comparison of Detection Results between rRT-PCR and HI Tests

In the middle school, amongst the 100 infectious cases confirmed by HI and/or rRT-PCR test, 22 were reported with positive rRT-PCR detection results and HI conversion, 26 were reported with positive rRT-PCR detection results and negative HI conversion, and 44 were reported with positive HI conversion without positive rRT-PCR detection results. A total of 8 cases were reported with positive rRT-PCR results but refused to take sero samples.

Of the 70 infection cases in the primary school, 28 were reported with positive rRT-PCR detection results and HI conversion, 21 were reported with positive rRT-PCR detection results and negative HI conversion, and 19 were reported with positive HI conversion without positive rRT-PCR detection results. 2 cases were reported with positive rRT-PCR results but refused to take sero samples. Given that a total of 10 individuals would not allow sero samples to be taken in the two schools, a maximum of 60 patients with proven infection may have sero converted across the two schools (50 confirmed and 10 unconfirmed). (Table 4 and Table S2).

**Table 4:** Comparison of detection results between rRT-PCR and HI test.

		HI conversion	
Field	rRT-PCR	Positive	Negative
Shandong	Positive	22	26
	Negative	44	0
Guizhou	Positive	28	21
	Negative	19	0

### The Factors Associated with Asymptomatic/symptomatic Infections

In the middle school, the median age of the 100 confirmed infectious students was 13 years old (ranging from 11 to 14 years old), and 53 (53.0%) were male. No students had underlying medical conditions or had received seasonal influenza vaccination or 2009 pandemic influenza vaccination during the past year. No student took oseltamivir or zanamivir before or after infection.

Two factors differed significantly amongst the symptomatic, asymptomatic and uninfected groups: the male/female ratio was higher in the symptomatic group (Table 5) than in the other two groups (p = 0.021), and the distribution of the three groups among the four classes was found to be different (p<0.001).

**Table 5:** Risk factors associated with symptomatic, asymptomatic and uninfectious groups*.

	Middle school in Shandong				Primary school in Guizhou			
	Group A (n = 29)	Group B (n = 71)	Group C (n = 89)	P Value	Group A (n = 27)	Group B (n = 43)	Group C (n = 50)	P Value
Median age (year, range)	12(11–14)	13(11–14)	13(11–15)	0.156	12(7–15)	11(6–14)	12(7–14)	0.832
Male/female ratio	2.6(21/8)	0.8(32/39)	0.8(39/50)	0.021	0.5(9/18)	0.6(16/27)	0.8(22/28)	0.639
BMI	17.5(14.0–21,1)	18.0(12.0–24.0)	17.2(12.6–21.8)	0.135	18.1(12.3–23.9)	18.1(11.0–25.2)	17.5(10.0–25.0)	0.631
Dormitory (yes/no)	29/0	70/1	84/5	0.259	17/10	29/14	31/19	0.852
Class distribution				<0.001				0.968
Class 1	13	40	18		8	15	16	
Class 2	2	7	31		13	17	21	
Class 3	12	16	16		6	11	13	
Class 4	2	8	24					

* Symptomatic cases are denoted Group A, asymptomatic are denoted Group B and uninfected are denoted Group C.

In the primary school, the median age of the 70 patients with H1N1 virus infection was 11 years old (ranging from 6 to 15 years old); and 25 (35.7%) were male. Among those pH1N1 infection patients, none had underlying medical conditions, no one had received seasonal or pandemic influenza vaccination in the previous 12 months, and no one took oseltamivir or zanamivir before or after infection. No difference was observed with regard to age, gender, BMI and distribution of classes in the infectious students with ARI symptoms, the asymptomatic infectious students and the non-infectious students. (Table 5).

## Discussion

During and after the H1N1 pandemic, estimates of the attack rate of the disease showed wide variability, depending on the detection methods used, the age group of the population and the periods of observation. Some studies have been conducted in schools and the results indicated that the attack rates of the H1N1 pandemic range from 4% to 42.4% [14], [15].

In the early stage of the pandemic, the H1N1 virus was associated with hundreds of fatal cases in Mexico [16], and it was found to spread widely in schools. As soon as H1N1 was reported in schools, closures were found to be necessary to reduce virus transmission and the risk of subsequent large outbreaks. However, as the pandemic progressed successive investigations suggested that, whilst infection was widespread in schools, severe illness did not generally occur [17]. During that time, it was hard to estimate the natural attack rate in the schools, because of school closure, the use of antiviral drugs as a preventive method and the introduction of vaccination.

In order to estimate the natural attack rate in the school, we selected two schools located in relatively isolated rural areas – few pandemic H1N1 cases had previously been reported in those areas and no preventative measures had been introduced in the schools. The attack rates of pandemic H1N1 in this study were estimated to be 58.3% and 52.9% in the primary and the middle school respectively. The rates were higher than in previous studies [6], [7], [12]. This may reflect the environmental conditions, the detection methods used and the duration of the studies. In this study all the students were tested by both HI and rRT-PCR, and the criteria for positive diagnosis were rRT-PCR positive or a 4 fold increase in HI titer. Previous studies have generally used either the rRT-PCR test or the HI test alone. Given that a proportion of rRT-PCR positive cases are found to be HI negative whilst a proportion of HI positive cases are found to be PCR negative, we conclude that previous studies that only use one of these tests may underestimate the true attack rate of the disease.

In our study, we combined rRT-PCR, a highly sensitive detection method, and HI assay, which helped us to screen as many infectious cases as possible (including asymptomatic cases [18]). The attack rate was estimated to be 33.7% using HI assay, and 29% using rRT-PCR. The use of rRT-PCR could detect only 62.9% positive samples successfully from all of the confirmed cases and HI assay could detect only 66.5% samples successfully. This result was consistent with Chinese National Influenza Centre’s (CNIC) previous reports [13], in which the attack rate of pH1N1 was 32.9% (95% CI: 30.2% to 35.6%) at age group 6–15 when using HI assay only.

Several cases in our study were found to be HI positive but PCR negative. One of the possible reasons may be related to the use of throat swabs rather than nasal swabs. Recent studies suggested that throat swabs are only able to detect around 82% of positive cases that are confirmed using nasal swabs [19]. However, Chinese influenza surveillance standards state that throat swabs must be carried out on children owing to the perceived invasiveness of nasal swabbing. Also, our study only lasted for 30 days so it is possible that some students may not have sero converted by the end of the study.

In this study, of all the 170 infectious subjects, a total of 56 students were reported to have symptoms, but only 43 patients were reported to have fever with an axillary temperature of ≥37.5°C. The asymptomatic infection rates were 35.8% and 37.6% in the primary school and the middle school respectively. The asymptomatic infection rates were 2 to 3 times higher than the symptomatic rates. The ratio of symptomatic infection to asymptomatic infection was 1∶2 whilst the ratio of the cases with fever to the cases without fever was around 1∶3. No hospitalizations or influenza-associated complications were found in either of the two schools. All of these values were consistent with previous studies [17], [20]. ILI and ARI set temperature ≥37.5°C as the cutoff point of fever, but asymptomatic infection seems lower than that, 37°C maybe a good cutoff point of fever in future study.

In our investigation, we found that the viral load of pandemic H1N1 was not significantly different between the symptomatically infectious patients and the asymptomatically infectious patients. The HI titer in the convalescence phase of the symptomatically infectious patients was similar to that of the asymptomatic infectious patients. Our data were consistent with previous studies that documented the time lines of influenza A viral shedding detected by rRT-PCR – peaks were found to occur around the same day as ARI symptoms onset before decreasing [21], [22]. Whereas some viral shedding was measured as early as two days before ARI onset, this occurred in a minority of cases. This means that the virus could potentially spread rapidly as soon as ARI symptoms occurred implying that the use of antiviral drugs may have been beneficial in reducing prevalence during the early stages of the outbreak. The different distributions of infectious students between classes in the middle school suggested that the virus would firstly spread within the class and then transmit to the other classes [6], [20].

## Materials and Methods

### Ethics

During this study, we adopted the procedures that were set out in the Law of the People’s Republic of China on Prevention and Treatment of Infectious Diseases (http://www.gov.cn/ziliao/flfg/2005-08/05/content_20946.htm). Participants were selected once verbal consent was received from the students themselves, their parents and their teachers and they were informed of their rights according to the law outlined above. During the study, all participants could withdraw at any time. We can confirm that all the data, including all questionnaires and samples, were gathered according to the ‘Program for novel A(H1N1) influenza surveillance’ and the ‘Guideline for ILI outbreak reports and investigation’, which were both issued by the Ministry of Health (MOH), China. No additional data were acquired by the authors.

### Study Design

The purpose of this study was to determine the attack rate and asymptomatic infection rate of H1N1 within schools. A cohort study was established. There are 31 administrative divisions of mainland China (22 provinces, 4 municipalities, 5 autonomous regions). 2 provinces (Shandong and Guizhou) were randomly selected to participate. One school was randomly selected from the schools that reported pH1N1 cases one week before the study in each province. In the investigation, we selected three classes randomly in each school. The middle school in Shandong had one class with ≥5 ILI cases at the beginning of the investigation, one class with fewer than 5 ILI cases, whilst the third class had no ILI cases. The primary school in Guizhou had no classes with ILI cases at the beginning of the investigation. In each school, the investigation started when the first confirmed case was found. The studies were organized by the Institute for Viral Disease Control and Prevention (IVDC), CDC, China. Each investigation team included one epidemiologist who was certificated by the Chinese Field Epidemiology Training Program (CFETP) and one laboratory professional staff from the IVDC. The same questionnaire was used in both schools, and all the interviewers were trained by the epidemiologist before the study. The throat swab and blood samples were collected by local CDC professional staff and monitored by IVDC staff. All swab and blood samples were stored in ice boxes and sent back to IVDC as soon as possible and all the laboratory tests (rRT-PCR and HI) were implemented by the same staff of IVDC.

### Survey

The survey was divided into general and individual-level sections. The general section included class-level questions (e.g., number of students in the class) and collective actions taken in response to new cases of the pandemic H1N1 outbreak at the school. Individual-level questions were also included in the questionnaire. Survey instructions directed the CDC experts to complete the survey for each member of the study population, consulting with the teachers as needed.

The local CDC decided to close the school only when one of the following three conditions was met: 1) a marked increase in hospitalizations; 2) influenza-associated complications occurred; 3) school operations were affected by absenteeism. During our study period, no school was closed. However, in the middle school, students with an axillary body temperature ≥37°C were asked to stay home for ≥7 days after onset of fever or feverishness. Students were only allowed to return to school after presenting a health certificate from the doctor. In the primary school, no student was isolated at home during the investigation.

The subjects were interviewed by questionnaire during the investigation. The questionnaire included: basic information, medical history, immunization history, the presence of ARI symptoms and personal contact situations.

### Study Population

In China, primary school students are generally 7–13 years old, whilst middle school students are 13–16 years old. However in remote areas such as those in this study, the primary school students were 5 to 15 years old whilst the middle school students were from 11 to 16 years old.

The middle school in Shandong had 3457 students enrolled in grades 6–9. The survey commenced on Nov. 22, 2009 and 214 students participated. The male/female ratio was 0.96, and the median age was 13 years old (ranging from 11 to 15).

The primary school in Guizhou had 796 students enrolled in pre-school and grades 1–6. The study population included 156 students. The male/female ratio was 0.79, and the median age was 12 years old (ranging from 6 to 15).

Of all those infected in the middle school in Shandong Province, 183 students stayed in dormitories and only 6 lived at home, whilst in the primary school in Guizhou province, 77 stayed in dormitories and 43 lived at home. In both schools, the dormitories were segregated by class, grade and gender.

The two schools provided uniform for all students and food for students who stayed in dormitories. Students were not forthcoming regarding their family status so we are unable to provide information regarding socio-economic status of the students in this study.

214 and 156 students participated in the interview and completed questionnaires in the middle school and primary school respectively. It was intended to collect blood samples from all the students during the course of this study. However, some students were absent owing to isolation at home, whilst others refused to participate in blood collection. Therefore, 2, 13, 11, 9, 21 and 6 swab samples were lost from the middle school on Nov. 24, 26, 28, 30 and Dec.10, 22 respectively whilst 16, 36 and 28 sero samples were lost on Nov. 24, Dec.10 and Dec.24 respectively. In the primary school, 3 swab samples were lost on Nov.26 and 28 swab samples on Dec.26, whilst one sero sample was lost on Nov.24 and Dec.10 and 28 sero samples were lost on Dec.26.

### Sample Collection and Detection Methods

The investigations were conducted in the middle school in Shandong Province from Nov. 22, 2009 and in the primary school in Guizhou Province from Nov. 26, 2009. During the investigation, all students were requested to measure their temperature in the morning before the class and the teachers were requested to measure the students’ temperature three times a day (morning, noon and evening). During the investigation, no case was identified, because all laboratory results were reported three months later. However, any student whose temperature was higher than 37°C was requested to stay at home for at least one week and return to school with a doctor’s health certificate. The blood samples were taken on Nov. 22, Dec. 6 and Dec. 22 in the middle school and on Nov. 26, Dec. 10 and Dec. 26 in the primary school. The swab samples were taken on the 1^st^, 3^rd^, 5^th^, 7^th^, 14^th^, 28^th^ days of the investigation. Wherever possible, blood and swab samples were taken in the school. However, in some cases (mainly for those students who were requested to stay at home), swab samples were taken at home or in the hospital.

The throat swab samples were detected by means of a quantitative reverse-transcriptase-polymerase-chain-reaction (rRT-PCR) assay to detect the presence of the H1N1 pandemic virus [23], [24]. The throat swab samples in a volume of 200 µL were prepared to RNA extraction using the RNeasy Mini Kit (Qiagen), and RNA extracted from the samples was eluted into 50 µL. Real-time PCR assays using AgPath one-step RT-PCR Kit (AB) was performed followed the manufacturer’s instructions. The selected primer and probe sets for identifying the pH1N1 included InFluA (F: 5′-GACCRATCCTGTCACCTCTGAC, R: 5′-GGGCATTYTGGACAA AKCGTCTACG, Probe: 5′-FAMTGCAGTCCTCGCTCA CTGGGCACG-BHQ1) and SWH1-1 (F: 5′-ACATTCGAAGCAACTGGAAA, R: 5′-GTRTTRCAATCGTG GAC TGG, Probe: 5′-FAM-TCCATTGCGAAKGCATATCTCGG-BHQ1).

To analyze the pH1N1 viral load in swabs, real-time RT-PCR was performed with a Strategene detection system using a fluorescently labeled TaqMan probe to enable continuous monitoring of amplicon formation. The primer and probe of Flu A M gene is from the WHO-released primer sets [25]. The primer and probe of house-keeping gene β-actin were obtained from the literature [26]. The concentration of primer and probe used was 40 mM and 10 mM, respectively. The reaction was completed in a total volume of 25 ml performed by QuantiTect Probe PCR Kit (Qiagen, Germany). The reaction mixture was incubated with 5 µl DNase-treated total RNA at the following temperature cycles. First, the reverse transcription reaction was completed by 1 cycle at 50°C for 30 min. Next, pH1N1 gene, and housekeeping (β-actin) genes were amplified by 1 cycle at 94°C for 15 min and 45 cycles at 94°C for 15 s, 55°C for 30 s, and 72°C for 30s each. As described in previous work [27], the standard curve was generated using serial dilution of in vitro transcribed standard RNA (10 to 10^7^ copies). The viral load level is presented as the ratio between copies of the target gene and β-actin gene.

Serum specimens were tested with a hemagglutination-inhibition (HI) assay for antibody responses to the H1N1 pandemic virus A/California/4/2009. The hemagglutination-inhibition (HI) assay using 0.5% Turkey red blood cells was used to test serum for antibody to pandemic H1N1 according to standard protocols [28], [29]. The 2009 pH1N1 antigen used was the A/California/07/2009 virus (provided by U.S. CDC), which was propagated in specific pathogen-free (SPF) embryonated chicken eggs and inactivated with 1% paraformaldehyde. A positive serum control (SPF chicken anti-serum against A/California/07/2009) and negative serum control (sera collected before the outbreak of pandemic H1N1) were included in each 96-well plate during the experiment. Prior to testing by the HI assay, serum samples were treated with a 1∶5 (vol/vol) of receptor destroying enzyme (RDE, prepared by CNIC) at 37°C for 18 hours followed by incubation at 56°C for 30 minutes. Serum samples were titrated in 2-fold dilutions in phosphate-buffered saline and tested at an initial dilution of 1∶10. Most individuals infected with influenza develop antibody titers ≥40 by viral HI assay after recovery and was therefore used as marker for immunity against pandemic H1N1 in this study.

### Definition

Influenza likely illness (ILI) was defined as fever (T≥37.5°C) with cough or sore throat. Acute respiratory illness (ARI) was defined as recent onset of at least two of the following: a) rhinorrhea or nasal congestion, b) sore throat, c) cough and d) fever or feverishness (T≥37.5°C).

A confirmed infectious case of pandemic H1N1 virus was defined as a person with laboratory confirmed infection at CNIC by one or more of the following tests: a) real-time RT-PCR or b) four-fold rise in HI titer. A symptomatic confirmed case was defined as a confirmed infection with ARI symptoms, and an asymptomatic infection was defined as confirmed infection without ARI symptoms.

### Analysis

The primary outcome of interest was confirmation of pH1N1 infectious cases in the study population. We calculated the attack rate as the percentage of students confirmed infectious with the H1N1 pandemic virus by rRT-PCR and/or HI test, and calculated the asymptomatic infection rate as the percentage of infectious students without typical ARI symptoms.

Statistical analysis was performed by the Chi square analysis, T-test, one-way ANOVA and Linear Regression analysis with Stata software (version 9).

## Supporting Information

Table S1 Number of students with confirmed pH1N1 showing symptoms.
(DOC)

Table S2 Detection results of rRT-PCR and HI array for each school by gender.
(DOC)

## References

[1] Ginsberg M, Hopkins J, Maroufi A, Dunne G, Sunega DR, et al (2009) Swine influenza A (H1N1) infection in two children–Southern California, March–April 2009. MMWR Morb Mortal Wkly Rep 58: 400–402. 19390508

[2] San Diego County Health and Human Svcs, Imperial County Public Health Dept,California Dept of Public Health, Dallas County Health and Human Svcs, Texas Dept of State Health Svcs, et al (2009) Update: swine influenza A (H1N1) infections–California and Texas, April 2009. MMWR Morb Mortal Wkly Rep 58: 435–437. 19407739

[3] Chan M (2009) World now at the start of 2009 influenza pandemic. WHO website. Available: http://www.who.int/mediacentre/news/statements/2009/h1n1_pandemic_phase6_20090611/en/. Accessed 2012 Sep 4.

[4] Chan M (2010) H1N1 in post-pandemic period. WHO website. Available: http://www.who.int/mediacentre/news/statements/2010/h1n1_vpc_20100810/en/. Accessed 2012 Sep 4.

[5] Lipsitch M, Hayden FG, Cowling BJ, Leung GM (2009) How to maintain surveillance for novel influenza A H1N1 when there are too many cases to count. Lancet 374: 1209–1211. 19679345 10.1016/S0140-6736(09)61377-5

[6] Lessler J, Reich NG, Cummings DA, Nair HP, Jordan HT, et al (2009) Outbreak of 2009 pandemic influenza A (H1N1) at a New York City school. N Engl J Med 361: 2628–2636. 20042754 10.1056/NEJMoa0906089

[7] Witkop CT, Duffy MR, Macias EA, Gibbons TF, Escobar JD, et al (2010) Novel Influenza A (H1N1) outbreak at the U.S. Air Force Academy: epidemiology and viral shedding duration. Am J Prev Med 38: 121–126. 19850440 10.1016/j.amepre.2009.10.005

[8] Miller E, Hoschler K, Hardelid P, Stanford E, Andrews N, et al (2010) Incidence of 2009 pandemic influenza A H1N1 infection in England: a cross-sectional serological study. Lancet 375: 1100–1108. 20096450 10.1016/S0140-6736(09)62126-7

[9] Donaldson LJ, Rutter PD, Ellis BM, Greaves FE, Mytton OT, et al (2009) Mortality from pandemic A/H1N1 2009 influenza in England: public health surveillance study. Bmj 339: b5213. 20007665 10.1136/bmj.b5213 PMC2791802

[10] Louie JK, Acosta M, Winter K, Jean C, Gavali S, et al (2009) Factors associated with death or hospitalization due to pandemic 2009 influenza A(H1N1) infection in California. Jama 302: 1896–1902. 19887665 10.1001/jama.2009.1583

[11] Jain S, Kamimoto L, Bramley AM, Schmitz AM, Benoit SR, et al (2009) Hospitalized patients with 2009 H1N1 influenza in the United States, April–June 2009. N Engl J Med 361: 1935–1944. 19815859 10.1056/NEJMoa0906695

[12] Ross T, Zimmer S, Burke D, Crevar C, Carter D, et al.. (2010) Seroprevalence Following the Second Wave of Pandemic 2009 H1N1 Influenza. PLoS Curr Influenza: RRN1148. 10.1371/currents.RRN1148 PMC2828126 20191082

[13] Xu C, Bai T, Iuliano AD, Wang M, Yang L, et al (2011) The seroprevalence of pandemic influenza H1N1 (2009) virus in China. PLoS One 6: e17919. 21533034 10.1371/journal.pone.0017919 PMC3080876

[14] Gurav YK, Pawar SD, Chadha MS, Potdar VA, Deshpande AS, et al (2010) Pandemic influenza A(H1N1) 2009 outbreak in a residential school at Panchgani, Maharashtra, India. Indian J Med Res 132: 67–71. 20693592

[15] Huai Y, Lin J, Varma JK, Peng Z, He J, et al (2010) A primary school outbreak of pandemic 2009 influenza A (H1N1) in China. Influenza Other Respi Viruses 4: 259–266. 10.1111/j.1750-2659.2010.00150.x PMC4634656 20795308

[16] Laguna-Torres VA, Benavides JG (2009) Infection and death from influenza A H1N1 virus in Mexico. Lancet 374: 2032–2033. 19913291 10.1016/S0140-6736(09)61916-4

[17] Cowling BJ, Chan KH, Fang VJ, Lau LL, So HC, et al (2010) Comparative epidemiology of pandemic and seasonal influenza A in households. N Engl J Med 362: 2175–2184. 20558368 10.1056/NEJMoa0911530 PMC4070281

[18] Lee N, Chan PK, Hui DS, Rainer TH, Wong E, et al (2009) Viral loads and duration of viral shedding in adult patients hospitalized with influenza. J Infect Dis 200: 492–500. 19591575 10.1086/600383 PMC7110250

[19] Zhang X, Yan HP, Ma YX, Wang Y, Zhao Y, et al (2011) Evaluation of nucleic acid amplification assay and rapid antigen assay of nasopharynx swabs and oropharynx swabs from glu-like patients in diagnosis of flu A. Chin J Infect Dis. 29: 154–157.

[20] Bautista E, Chotpitayasunondh T, Gao Z, Harper SA, Shaw M, et al (2010) Clinical aspects of pandemic 2009 influenza A (H1N1) virus infection. N Engl J Med 362: 1708–1719. 20445182 10.1056/NEJMra1000449

[21] Lau LL, Cowling BJ, Fang VJ, Chan KH, Lau EH, et al (2010) Viral shedding and clinical illness in naturally acquired influenza virus infections. J Infect Dis 201: 1509–1516. 20377412 10.1086/652241 PMC3060408

[22] Carrat F, Vergu E, Ferguson NM, Lemaitre M, Cauchemez S, et al (2008) Time lines of infection and disease in human influenza: a review of volunteer challenge studies. Am J Epidemiol 167: 775–785. 18230677 10.1093/aje/kwm375

[23] Wang W, Pan M, Chang GH, Li XD, Li TS, et al.. (2009) Laboratory Confirmation of the First Influenza A(H1N1) Imported Case in Mainland China. Chinese Journal of Virology 25 4–7. 20361591

[24] WHO CDC protocol of real-time RT-PCR for influenza A(H1N1).

[25] World health organization (2005) Recommended laboratory tests to identify avian influenza A virus in specimens from humans. WHO website. Available: http://www.who.int/csr/disease/avian_influenza/guidelines/avian_labtests2.pdf. Accessed 2012 Sep 4.

[26] Zhou J, Law HK, Cheung CY, Ng IH, Peiris JS, et al (2006) Differential Expression of Chemokines and Their Receptors in Adult and Neonatal Macrophages Infected with Human or Avian Influenza Viruses. J Infect Dis194(1): 61–70. 10.1086/504690 PMC7110244 16741883

[27] Di Trani L, Bedini B, Donatelli I, Campitelli L, Chiappini B, et al (2006) A sensitive one-step real-time PCR for detection of avian influenza viruses using a MGB probe and an internal positive control. BMC Infect Dis 6: 87–94. 16725022 10.1186/1471-2334-6-87 PMC1524785

[28] Kendal AP, Skehel JJ, Pereira MS (1982) World Health Organization Collaborating Centers from Reference and Research on Influenza: concepts and procedures for laboratory-based influenza surveillance, pB 17–35.

[29] WHO, WHO Manual on animal influenza diagnosis and surveillance 2002.5. WHO website. Available: http://www.who.int/vaccine_research/diseases/influenza/WHO_manual_on_animal-diagnosis_and_surveillance_2002_5.pdf. Accessed 2012 Sep 4.

