# Supplementary material for: Epidemiological and Virological Characteristics of Pandemic Influenza A (H1N1) School Outbreaks in China in 2009
Source: PLoS One. 2012 Sep 27;7(9):e45898. doi: 10.1371/journal.pone.0045898 (PMC3459944; doi:10.1371/journal.pone.0045898)
Supplement: Table S1 — Number of students with confirmed pH1N1 showing symptoms. (DOC) [file pone.0045898.s001.doc]

**Table S1. Number of students with confirmed pH1N1 showing symptoms**

| Symptoms | Shandong | Guizhou |
| --- | --- | --- |
| fever | 29(29%) | 13(19%)* |
| cough | 21(21%) | 34(49%) |
| sore throat | 13(13%) | 14(20%) |
| stuffy | 12(12%) | 11(16%) |
| runny | 16(16%) | 30(43%) |
| * all recorded temperatures are lower than 37.5℃ | | |
