# Supplementary material for: Epidemiological and Virological Characteristics of Pandemic Influenza A (H1N1) School Outbreaks in China in 2009
Source: PLoS One. 2012 Sep 27;7(9):e45898. doi: 10.1371/journal.pone.0045898 (PMC3459944; doi:10.1371/journal.pone.0045898)
Supplement: Table S2 — Detection results of rRT-PCR and HI array for each school by gender. (DOC) [file pone.0045898.s002.doc]

**Table S2.** **Detection results of rRT-PCR and HI array for each school by gender**

|  |  | PCR | | HI* | |
| --- | --- | --- | --- | --- | --- |
| Location | Gender | - | + | - | + |
| Shandong | male | 87 | 19 | 59 | 31 |
|  | female | 94 | 14 | 59 | 34 |
| Guizhou | male | 51 | 19 | 36 | 18 |
|  | female | 54 | 32 | 50 | 29 |

* 54 missing in HI test
